# Cardiomyopathy in a c.1528G>C *Hadha* mouse is associated with cardiac tissue lipotoxicity and altered cardiolipin species

**DOI:** 10.1016/j.jlr.2025.100792

**Published:** 2025-03-29

**Authors:** Chibuike Eke, Shannon Babcock, Garen Gaston, Gabriela Elizondo, Hak Chung, Ayah Asal, Kathryn C. Chatfield, Genevieve C. Sparagna, Andrea E. DeBarber, William Packwood, Jonathan R. Lindner, Melanie B. Gillingham

**Affiliations:** 1 Department of Molecular and Medical Genetics, Oregon Health and Science University, Portland, Oregon, USA; 2 Department of Pediatrics, University of Colorado Anschutz Medical Campus, Children's Hospital Colorado, Aurora, Colorado, USA; 3 Division of Cardiology, Department of Medicine, University of Colorado Anschutz Medical Campus, Aurora, Colorado, USA; 4 Department of Chemical Physiology & Biochemistry, Oregon Health and Science University, Portland, Oregon, USA; 5 Knight Cardiovascular Institute, Oregon Health and Science University, Portland, Oregon, USA; 6 Cardiovascular Division, The University of Virginia Medical Center, Charlottesville, Virginia, USA

**Keywords:** Lipids/oxidation, lipolysis and fatty acid metabolism, oxidized lipids, lipid droplets, mitochondria, phospholipids, fatty acid oxidation, cardiolipin

## Abstract

Long-chain 3-hydroxyacyl-CoA dehydrogenase deficiency (LCHADD) is a metabolic disorder caused by the loss of LCHAD enzymatic activity in the α-subunit of the trifunctional protein (TFPα), leading to impaired fatty acid oxidation (FAO). Patients with LCHADD often develop dilated cardiomyopathy. A previously unrecognized enzymatic function of TFPα as monolysocardiolipin acyltransferase (MLCL-AT) has been implicated in cardiolipin remodeling, crucial for mitochondrial cristae integrity. However, it remains unclear whether the common pathogenic variant c.1528G>C in *HADHA* impairs MLCL-AT activity in TFPα. In this study, we investigated whether cardiac cardiolipin profiles are altered in LCHADD and explored potential pathophysiological mechanisms, including heart lipid accumulation, changes in the cardiolipin synthesis pathway, and mitochondrial dynamics, utilizing a murine model of LCHADD carrying c.1528G>C variant that mimics the cardiomyopathy observed in humans. LCHADD mice developed eccentric hypertrophic cardiomyopathy from 3- to 12 months of age. 12-month-old LCHADD hearts exhibited altered cardiolipin profiles and increased oxidized cardiolipin. LCHADD hearts had higher lipid content, and the shift in fatty acid profile mirrored the shift in cardiolipin profile compared to wild-type controls, suggesting altered cardiolipin composition in LCHADD may be a reflection of accumulated lipids caused by lower FAO. No differential expression of cardiolipin synthesis and remodeling pathway enzymes was observed, suggesting minimal impact of the c.1528G>C variant on cardiolipin remodeling pathway. LCHADD hearts showed an altered ratio of OPA1 isoforms, and mitochondria with swelling and disorganized cristae were present. These findings suggest that altered fatty acid, cardiolipin profiles, and mitochondrial dynamics may contribute to LCHADD cardiomyopathy, warranting further studies.

## Graphical Abstract

**Figure:**
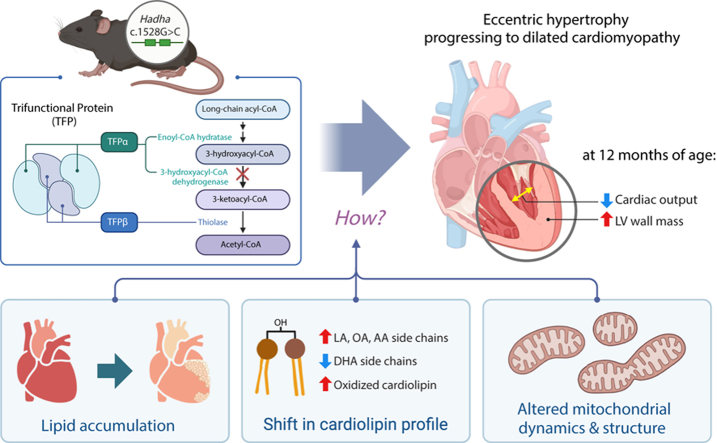

Long-Chain 3-Hydroxyacyl-CoA Dehydrogenase Deficiency (LCHADD, OMIM #609016) is a rare inherited metabolic disorder that disrupts the β-oxidation of long-chain fatty acids. This disorder leads to a range of clinical manifestations, such as hypoketotic hypoglycemia, hepatomegaly, encephalopathy, episodic rhabdomyolysis, exercise intolerance, and peripheral neuropathy (1). Patients may also develop dilated cardiomyopathy, characterized by the enlargement of the left ventricle (LV) and LV systolic dysfunction, which is a frequent cause of mortality in LCHADD patients (1, 2).

The most common LCHADD pathogenic variant is c.1528G>C in *HADHA.* At the molecular level, *HADHA* plays a pivotal role in long-chain fatty acid oxidation (FAO) by encoding the α-subunit of mitochondrial trifunctional protein (TFPα), one of two subunits that comprise the TFP complex. Located on the inner mitochondrial membrane, TFP carries out the final steps of β-oxidation of long-chain fatty acids with three enzymatic activities: long-chain enoyl-hydratase, long-chain 3-hydroxyacyl-CoA dehydrogenase (LCHAD), and long-chain 3-ketothiolase (3). The common c.1528G>C pathogenic variant in *HADHA* specifically reduces the LCHAD activity of TFP while leaving the other two enzymatic activities relatively unaffected (4).

Patients with long-chain FAO disorders, including LCHADD, frequently present with cardiomyopathy in infancy, highlighting the importance of FAO to cardiac function (5, 6, 7, 8). Patients may initially present with cardiac hypertrophy that can develop into dilated cardiomyopathy and heart failure or cardiac arrhythmias (5). Recent evidence also suggests that cardiac complications can re-emerge later in life (9). While the exact etiology is not known, hypotheses revolve around energy deficit or lipid toxicity. Cardiac tissue relies heavily on FAO to meet its metabolic demands. In the absence of FAO, alternate energy substrates, such as glucose and ketones, may be insufficient in fueling the heart, especially during times of exertion; therefore, the loss of FAO may create an energy deficiency, leading to cardiomyopathy (10, 11). Alternatively, FAO defects may cause a buildup of wholly unprocessed lipids or partially processed lipids, either of which may be toxic to the cells and contribute to or cause cardiac dysfunction (12, 13).

A separate enzymatic functionality recently found for TFPα suggests a new hypothesis. TFPα and its possible isoforms were shown to have monolysocardiolipin acyl transferase (MLCL-AT) activity. MLCL-AT is involved in the remodeling of cardiolipin (14, 15), a unique diphosphatidylglycerol lipid located primarily in the mitochondrial membrane (16). Cardiolipin plays a key role in maintaining mitochondrial health through the regulation of mitophagy, the formation of inner membrane cristae, and the localization of enzymes involved in the electron transport chain for optimal bioenergetics (17, 18, 19, 20). The identification of MLCL-AT activity of TFPα raises the possibility of an LCHADD-specific pathogenic mechanism for cardiomyopathy. Altered cardiolipin biosynthesis/remodeling may impact the mitochondrial function and dynamics, leading to cardiac dysfunction. This hypothesis is supported by the example of Barth syndrome, an X-linked disorder that manifests cardiomyopathy. It is caused by mutations in the *TAFAZZIN* gene, which encodes a key enzyme in the cardiolipin remodeling pathway (21, 22).

It is not known whether the c.1528G>C pathogenic variant in *HADHA* affects MLCL-AT activity and cardiolipin remodeling. However, it has been speculated from crystal structure analysis that this variant may eliminate the MLCL-AT activity (23). Additionally, human iPSC-derived cardiomyocytes with the c.1528G>C variant show an altered cardiolipin profile versus unaffected control (24, 25). Research has been limited on the molecular pathogenesis of LCHADD cardiomyopathy in part due to the lack of adequate animal models. Our recently developed c.1528G>C knock-in mouse line develops a hypertrophic cardiomyopathy phenotype, mirroring the salient pathophysiological characteristics seen in patients with LCHADD, thus offering a valuable tool to study the underlying pathophysiological mechanisms of the disease (26). Using this model, this study aims to further characterize the cardiomyopathy and to investigate the molecular mechanisms involved. Specifically, we aim to address how the c.1528G>C variant alters lipid and cardiolipin profiles in the heart and look into the effects on the cardiolipin synthesis pathway and mitochondrial dynamics.

## Materials and Methods

### c.1528G>C *Hadha* mouse model

All animal procedures were reviewed and approved by the Oregon Health and Science University IACUC (eIACUC #B11243). The mouse strain was created by introducing the target mutation, c.1528G>C, along with a silent mutation to prevent recutting, into exon 15 of *Hadha* through homology-directed repair using CRISPR/Cas9 as previously described (26). Mice are on a C57BL/6 background.

### Echocardiography

High-frequency (40 MHz) two-dimensional echocardiography was performed to assess the status of the LV (Vevo 2100, VisualSonics). End-systolic and end-diastolic LV dimensions and wall thickness, and LV ejection fraction were measured from the parasternal long-axis view by the single plane modified Simpson’s method. Stroke volume (SV) was calculated as the difference between end-diastolic and end-systolic LV volumes calculated by Equation 1:

(1) LVvol=5/6×EndMaj×EndArea

where LV vol = Left Ventricular Volume (mL), EndMaj = Endocardial Major axis (mm), and EndArea = Endocardial Area (mm^2^).

Left ventricular mass was calculated by end-diastolic images in the mid-ventricular parasternal short-axis view by Equation 2:

(2) $$\text{LV}\;\text{mass}=1.05\times \left(5/6A_{1}\left[l+t\right]-5/6A_{2}\left[l\right]\right)$$

where *A*_*1*_ and *A*_*2*_ are the cross-sectional areas for the epicardium and endocardium, respectively; *l* is the distance from the apical endocardium to the mitral valve plane; and *t* is mean wall thickness.

Cardiac output was calculated as SV × heart rate (HR).

### Lipidomic cardiolipin analysis

Heart tissue was homogenized using a glass-on-glass homogenizer in PBS, and lipids were extracted as detailed previously, and cardiolipin was quantified in these lipid extracts using liquid chromatography coupled to electrospray ionization mass spectrometry (LC-MS) in an API 4000 mass spectrometer (Sciex) using normal-phase solvents (27). Tetramyristal cardiolipin as an internal standard and tetraoleoyl cardiolipin as a reference standard (both from Avanti Polar Lipids, Birmingham, AL) were used for a standard curve to quantify amounts in nmol/mg (27). Total cardiolipin was calculated as the sum of 19 dominant species and MLCL as the sum of 9 species. Oxidized species of cardiolipin containing 4 linoleate chains (72:8) or three linoleates plus one oleate (72:7) were measured by detection of peaks at *m/z* 1464 and *m/z* 1466, respectively, with retention time identical to the non-oxidized cardiolipin species.

### Fatty acid oxidation activity assay

FAO enzyme activity was quantified using the Long Chain Fatty Acid Oxidation Assay Kit (Biomedical Research Service, E-141L) per manufacturer's protocol. Snap-frozen cardiac tissue samples were homogenized in a provided Sample Buffer using a Bead Mill homogenizer. After centrifugation, supernatants of the lysates were normalized to the concentration of 0.5 mg protein/ml Sample Buffer. 20 μl of prepped sample was mixed with FAO Assay Solution with palmitoyl-CoA as FAO substrate. Samples without FAO substrate were included as a control. Plates were placed in a 37°C humidified incubator for 30 min, and the optical density at 492 nm was measured. FAO enzyme activity was calculated using respective controls and presented as relative to WT.

### RNA extraction, cDNA synthesis, and quantitative PCR (qPCR)

Total RNA was extracted from snap-frozen cardiac tissue using RNeasy Fibrous Tissue Mini Kit (Qiagen, 74704) following the manufacturer's instructions. An in-column DNase digestion step was included to eliminate potential genomic DNA contamination. cDNA was generated using a High-Capacity RNA-to-cDNA Kit (Thermo Fisher Scientific, 4387406) in accordance with the provided protocol.

qPCR was performed with QuantStudio 5 Real-Time PCR System (Thermo Fisher Scientific, A34322) and Power Sybr Green PCR Master Mix (Thermo Fisher Scientific, 4367659). The primer sequences used are provided in supplemental Table S1. Primers for *Cds1* and *Crls1* were previously published (28, 29). The primers were meticulously designed to span two exons of the target genes.

### Western blot analysis

Snap-frozen cardiac tissue samples were homogenized and sonicated in ice-cold RIPA lysis buffer with protease inhibitors (Santa Cruz Biotechnology, sc-24948). Total protein was quantified using Pierce™ BCA protein Assay Kit (Thermo Scientific, A55864). Equal amount of protein samples (∼20–30 μg) were separated by SDS-PAGE employing TGX Stain-Free gels (Bio-Rad, 456-8036) and transferred onto PVDF membranes. The blots were probed with relevant primary antibodies and matched secondary antibodies for an hour at room temperature or overnight at 4°C. Antibody information and concentrations are listed in supplemental Table S2. Target protein bands were visualized with the PicoPlus HRP Chemiluminescence kit (Thermo Fisher, 34580) using an Azure Sapphire imager. A Precision Plus Dual Color Standard (Bio-Rad, 1610374) aided in size estimation. Total protein was visualized on a GelDoc EZ Imager (Bio-Rad, 1708270). The densitometric values for bands and total protein were analyzed on the Bio-Rad Image Lab 6.1.

### Fatty acid methyl ester (FAME) assay

#### Chemicals and Reagents

The 20 organic acid reference standards and 3 internal standard compounds used were purchased from Nu-Chek Prep Inc. Hexane and methanol solvents, along with general lab supplies such as vials, caps, and inserts, were from VWR. Acetyl chloride was from Sigma-Aldrich.

#### Preparation of Calibrators/Samples

Frozen cardiac tissue was processed for the FAME assay to quantify fatty acids in cardiac tissue. Working solutions of 20 organic acids were prepared in methanol for use as calibrators for the assay. Heart tissue samples were transferred to 2 ml bead beater tubes containing 1.4 mm ceramic beads (Fisher Scientific). PBS (Gibco, Grand Island, NY) was added to the tubes at 25 mg/ml wet weight/volume ratio and samples were homogenized using a BeadBlaster 24R device (settings: speed 3650 rpm, linear speed 6 m/s, cycle time 30 s, rest cycle 30 s, cycles 2, temp 4°C). Mouse Serum (20 μl) or heart tissue sample homogenates (50 μl) or reference standard mix (various volumes) and 5 μl internal standard mixture (undecanoic acid 80 ng/μl, heptadecanoic acid 800 ng/μl and heneicosanoic acid 800 ng/μl) were mixed in screw-capped glass tubes and 2 ml of methylation mixture was added (methanol/acetyl chloride, 20:1 v/v). The mixture was incubated at 25°C with shaking at 100 rpm for at least 12 h. After incubation, methyl esters were isolated using liquid-liquid extraction performed by adding 0.5 ml water and 1.0 ml hexane to each sample tube. The mixture was vortexed for 20 s and centrifuged for 5 min at 2,000 rpm. The upper (organic) layer was removed and placed into an auto-sampler vial for analysis using gas chromatography-mass spectrometry (GC-MS).

#### GC-MS analysis

The analysis was performed using an Agilent 7890B GC with a 5977A MS detector with an autosampler and split/splitless injector operated in split mode. The column was an Agilent DB-Fast FAME column (30 m, 0.25 mm id, 0.25 μm film thickness). Helium was the carrier gas at a flow rate of 1 ml/min. The injection port and auxiliary heater were maintained at 250°C. A 1 μl sample was injected in split mode (1:10) at an initial oven temperature of 50°C, held for 0.5 min, and then increased at 25°C/min to 194°C, held for 1 min followed by 5°C/min to 245°C, held for 3 min and then returned to 50°C. The MS was operated at a source temperature of 220°C and an MS quad temperature of 150°C in positive electron impact mode. Fatty acid methyl esters were detected by selected ion monitoring of ions at *m/*z 55, 67, 74, 79, and 91, each with a 25 msec dwell time after a solvent delay of 4 min. The GC-MS was controlled, and data were acquired using enhanced MassHunter software version B.07.04.2260, and results were analyzed using Agilent MassHunter quantitative analysis software version B.07.0. Calibration curves for quantification were generated from peak area ratios for authentic standard: internal standard for calibrator samples.

### Triglyceride extraction and quantification

20 mg of snap-frozen cardiac tissue was homogenized in 100 μl extraction buffer containing 5 volumes of isopropanol, 2 volumes of Milli-Q water, and 2 volumes of Triton X-100. After centrifugation and 30-min incubation at room temperature, triglyceride content was measured with Triglyceride Assay Kit (EnzyChrom™, ETGA-200) per manufacturer's protocols.

### Heart sectioning and Oil Red O staining

Hearts were dissected from mice sacrificed by isoflurane inhalation followed by cervical dislocation. Ventricles were placed in buffered formalin overnight and then transferred to 30% sucrose until sectioning. Ventricles were embedded in optimal cutting temperature (OCT) compound and sectioned into 8 μm slices using a Tanner Scientific TN50 Cryostat Microtome. All WT and LCHADD tissues were stained in same batch for direct comparison. Slides were placed in 60% isopropanol for 3–5 min followed by 20 min in a fresh, filtered Oil Red O working solution in 60% isopropanol. Slides were briefly cleaned in 60% isopropanol then rinsed in water and counterstained with Hematoxylin Stain-Plus (Fisher). Images were obtained using KEYENCE® BZ-X800 microscope.

### Transmission electron microscopy (TEM)

Mice were perfused quickly with PBS and then switched to 2.5% glutaraldehyde and 2.0% formaldehyde in 0.1 M PBS, pH 7.4. After perfusion, the cardiac LV was dissected and processed in a Pelco Biowave Pro+ microwave with a SteadyTemp Pro chiller. The tissue was rinsed and stained with 2% osmium tetroxide and 1.5% potassium ferricyanide, followed by 1% uranyl acetate. The tissue was dehydrated in an increasing acetone series followed by infiltration in EMbed812 resin. The tissue was flat-embedded in coffin molds and polymerized in a 60°C oven. Once polymerized, the capsules were removed and sectioned. Sections were collected on slot grids and imaged on a Tecnai T12 transmission electron microscope equipped with an AMT Nanosprint12 camera, at 120 kV.

Six images from each animal were randomly selected and assigned to four blinded reviewers. Images were divided into octants. Mitochondria from two or more octants per image were outlined and analyzed using FIJI software. Area was calculated in each octant and the entire image using only cardiomyocyte fiber area and subtracting nucei area. Events of cristolysis were registered by each reviewer over an entire image and applied to the calculated number of mitochondria in the image.

### Mitochondrial copy number

Cardiac DNA was isolated using QIAamp DNA Mini Kit (Qiagen, 51304). The isolated DNA was used to perform qPCR on the QuantStudio 5 Real-Time PCR System (Thermofisher) with ∼10 ng of DNA, 250 nM of forward/reverse *mt-Nd6* or *Polb* primers (sequences provided in supplemental Table S3), and Power Sybr Green PCR Master Mix (Thermofisher). The relative copy number was calculated as previously published (30).

### Statistical analysis

All statistical analyses and graphical representations were conducted using GraphPad Prism (Version 10.0.2, GraphPad Software, San Diego, CA) or Biorender (biorender.com). Data were graphed as the mean ± standard deviation of the mean (SD) with individual data points. *P* < 0.05 was considered statistically significant.

Differences between LCHADD and WT mice by sex (male and female) were compared by a 2-way ANOVA with a post-hoc Sidak multiple comparison test for echocardiography outcomes. Tissue cardiolipin species, plasma fatty acids, and tissue fatty acids were compared by 2-way ANOVA (main effects: genotype and cardiolipin or fatty acid species) with a post-hoc Sidak multiple comparison test. Total cardiolipin, MLCL/CL, total MLCL, and oxidized cardiolipin species differences between LCHADD and WT mice by sex (male and female) were compared by a 2-way ANOVA with a post-hoc Sidak multiple comparison test. RT-qPCR and immunofluorescence results were compared by unpaired *t* test between genotypes (LCHADD and WT) by sex. Western blot protein densitometry was normalized to GAPDH and compared by a 2-way ANOVA (genotype and sex) with a post-hoc Sidak multiple comparison test. Heart mitochondrial copy number was compared by unpaired *t* test between LCHADD and WT. Mitochondrial TEM data was compared by nested, two-tailed *t* test.

## Results

### Progressive hypertrophic cardiomyopathy

We have previously reported the development of hypertrophic cardiomyopathy in 12-month-old LCHADD mice (26). Other murine models of FAO disorders have described variable cardiac phenotypes including either a non-progressive hypertrophic cardiomyopathy that does not change with age or a progressive hypertrophic cardiomyopathy that worsens over time (31). To further characterize the cardiac phenotype in LCHADD mice, we performed echocardiography in WT and LCHADD male and female mice at 3 months of age and compared the cardiac parameters to our previous results. In old and young LCHADD males compared to their WT counterparts, SV, cardiac output, and ejection fraction were significantly or trended to be lower (Table 1). In young LCHADD male mice, however, this was due primarily to a reduced diastolic LV volume, consistent with concentric remodeling. By 12 months, LCHADD males diastolic LV volume was similar to WT; however, LV mass increased along with decreased cardiac output, which is consistent with eccentric hypertrophic cardiomyopathy. While there was no statistical difference in echocardiography between female LCHADD and WT mice at 3 months of age by 2-way ANOVA, a similar pattern of lower SV and cardiac output among 3-month-old female LCHADD mice was observed. Female LCHADD mice had a similar eccentric hypertrophy by 12 months of age (Table 1). The LCHADD mouse model showed a progressive cardiac phenotype initially characterized by concentric remodeling that progresses to eccentric hypertrophic cardiomyopathy.

**Table 1:** Echocardiogram changes from 3 to 12 months of age in LCHADD and WT mice

Cardiac parameters	Male						Female					
	LCHADD			WT			LCHADD			WT		
	3 months n = 6	12 months n = 14	Diff	3 months n = 6	12 months n = 12	Diff	3 months n = 6	12 months n = 11	Diff	3 months n = 6	12 months n = 14	Diff
Heart Rate (bpm)	462 ± 25	474 ± 34	12	466 ± 32	499 ± 31	34	424 ± 34	463 ± 37	39	423 ± 73	480 ± 44	45
LV systolic volume (μl)	22.4 ± 2.0	33.6 ± 9.4	11.2	31.1 ± 12.7	33.9 ± 15.0	2.8	19.9 ± 5.0	23.5 ± 6.5	3.6	18.6 ± 5.3	21.7 ± 4.9	3.1
LV diastolic volume (μl)	45.7 ± 3.6∗∗∗	65.1 ± 12.0	19.4	70.1 ± 18.1	75.7 ± 20.7	5.6	42.8 ± 3.6	46.9 ± 7.7	4.1	47.6 ± 10.1	51.9 ± 8.2	4.2
Stroke Volume (μl)	23.3 ± 4.7∗	31.5 ± 6.1^a^	8.2	39.0 ± 6.1	41.8 ± 8.4	2.8	22.9 ± 1.9	23.4 ± 5.1^a^	0.6	29.1 ± 5.3	30.2 ± 5.4	1.1
Cardiac Output (ml/min)	10.7 ± 1.9∗∗∗	15.0 ± 3.4^a^	4.3	18.2 ± 3.4	20.8 ± 3.7	2.6	9.7 ± 1.3	10.8 ± 2.5^a^	1.1	12.4 ± 1.0	14.5 ± 3.0	2.1
Ejection Fraction (%)	50.6 ± 6.7	48.9 ± 6.5^a^	−1.7	56.9 ± 6.6	56.5 ± 7.3	−0.4	54.0 ± 8.0	50.3 ± 9.3^a^	−3.8	61.5 ± 4.5	58.3 ± 5.8	−3.2
LV mass (mg)	104.7 ± 12.1	133.4 ± 13.7^a^	28.7	98.6 ± 14.6	110.0 ± 20.8	11.5	82.5 ± 9.3	103.1 ± 12.2^a^	20.6	78.6 ± 9.5	87.3 ± 12.1	8.4

Data are mean ± SD and compared by 2-way ANOVA (genotype × time) by sex. ∗(*P* < 0.05), ∗∗∗(*P* < 0.001) diff = difference from 3 months to 12 months; mo = month-old.

a Denotes data from previous publication (26).

### Fatty acid oxidation: Expression, functionality, and lipid accumulation

**Fig. 1:**
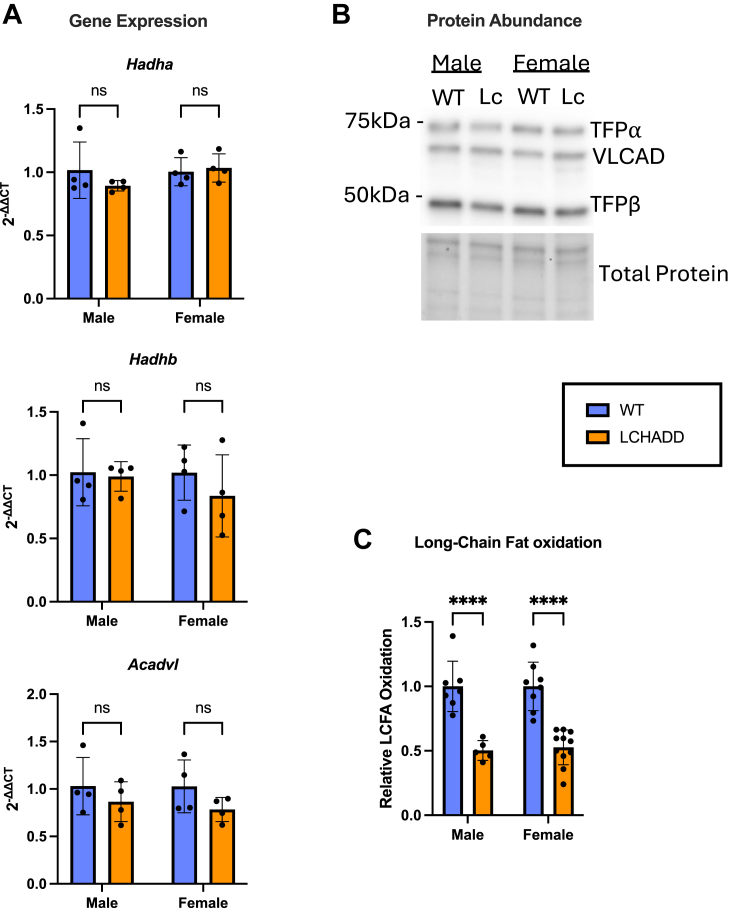
**FAO enzyme expression, and function in LCHADD hearts**. A: mRNA levels of *Hadha*, *Hadhb*, *Acadvl* (N = 4 per group) and (B) protein levels of TFPα, TFPβ and VLCAD (pooled samples, N = 3 per group) are comparable in the cardiac tissue of LCHADD and WT mice. C: LCHADD mice have reduced long-chain fatty acid (LCFA) oxidation in the heart (WT male N = 7, WT female N = 8, LCHADD male N = 5, LCHADD Female N = 11). Data presented as mean ± SD with individual data points. Statistics: (A) 2-way ANOVA (genotype & sex) or (C) *t* test. ns (not significant), ∗∗∗∗ (*P* < 0.0001).

First, we verified our previous findings that the cardiac tissue of LCHADD mice maintains similar FAO gene and protein expression but with lower FAO capacity (25). In this report, we confirmed that mRNA levels of FAO genes (*Acadvl*, *Hadha*, and *Hadhb*) and protein levels of the respective proteins (VLCAD, TFPα, and TFPβ) are similar between genotypes (Fig. 1A, B and supplemental Fig. S3). We also found FAO capacity of LCHADD hearts is indeed reduced compared to WT hearts using an alternative, non-radioactive method (Fig. 1C).

### Analysis of cardiolipin composition in the cardiac tissue of LCHADD mice

**Fig. 2:**
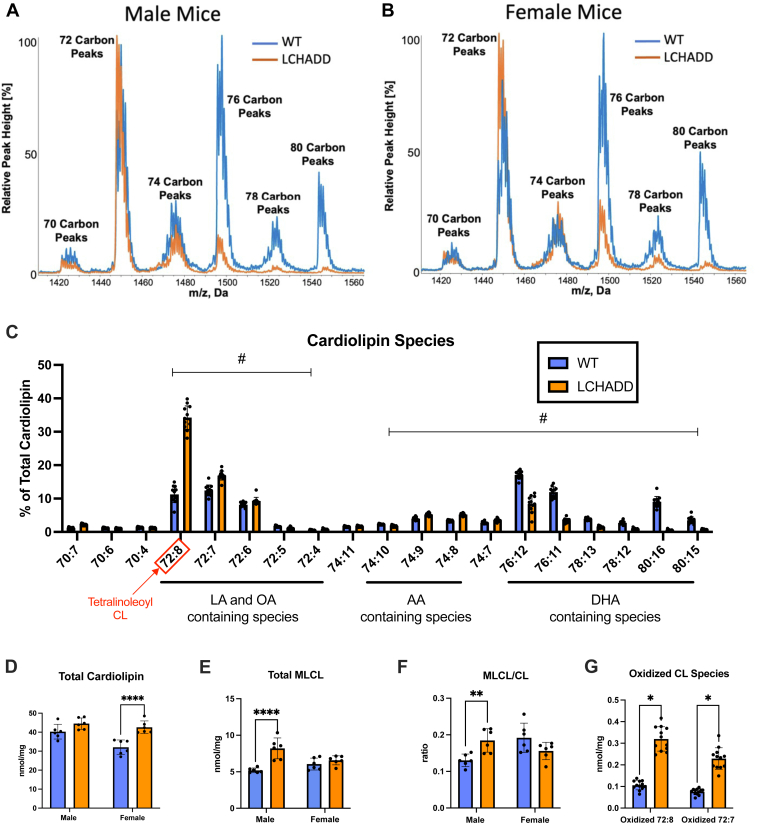
**Cardiac cardiolipin profiles in 12-month-old LCHADD mice differ from WT**. A, B: Representative mass spectra of cardiolipin species in WT (blue trace) and LCHADD (orange trace) in male (A) and female (B) cardiac tissue illustrating alterations in cardiolipin species due to the LCHADD genotype. C: Relative abundance of individual cardiolipin (CL) species in the heart identified by their ratio of carbons to double bonds. Species with side chains predominant in oleate (OA), linoleate (LA), arachidonate (AA), or docosahexaenoate (DHA) are indicated. Species that exhibit significant differences of *P* < 0.005 between WT and LCHADD mice are collectively marked with #. D–F: Sex differences can be seen in total cardiolipin (sum of 19 species) (D), total monolysocardiolipin (MLCL) (sum of 9 species) (E), and MLCL/CL ratio (F). G: Oxidized CL showing species 72:7 and 72:8 (tetralinoleoyl), which have 3- and 4-linoleoyl side chains, respectively. For (C–G), N for each genotype: females N = 6, male N = 6, combined N = 12. Bar graphs represent mean ± SD with individual data points. Statistics: individual cardiolipin species (C) were analyzed by multiple *t* test with a two-stage false discovery rate. Other data (D–G) was analyzed by 2-way ANOVA with a post-hoc Sidak test. Significant differences are marked with a # (*P* < 0.005) or denoted by asterisks: ∗ (*P* < 0.05), ∗∗ (*P* < 0.01), ∗∗∗∗ (*P* < 0.0001).

TFPα′s non-canonical role as MLCL-AT opens up a new avenue to be explored in regard to potential mechanism of cardiomyopathy development in LCHADD, as altered cardiolipin has been shown to affect cardiac function (17, 18, 19, 20). To look into this possibility, we analyzed cardiolipin profiles in cardiac tissue from 12-month-old WT and LCHADD mice using LC-MS (Fig. 2). As previously reported, the predominant cardiolipin species in human and rat hearts is tetralinoleoyl cardiolipin, a species with four linoleate side chains (32, 33). However, in mouse hearts, depending upon the strain and perhaps diet, the profile of cardiolipin varies widely and incorporates various alternate fatty acyl side chain species into the cardiolipin molecule (34). Our WT male and female cardiac tissue had a predominant peak at cardiolipin sizes, having a total number of side chain carbons at 72 but also at 76 carbons with lower peaks at 70, 74, 78, and 80 carbons (Fig. 2A, B). In comparison, peaks at 76, 78, and 80 carbons were all markedly reduced in LCHADD hearts. Specifically, LCHADD mice had increases in the percentage of species having linoleate (LA), oleate (OA), arachidonate (AA), and other 20 carbon side chains, while the species containing docosahexaenoate (DHA) and other 22 carbon species were significantly reduced (Fig. 2C). Total cardiolipin levels and MLCL levels were also measured and showed unexpected sex-specific differences. LCHADD females but not males showed higher total cardiolipin levels compared to WT (Fig. 2D). LCHADD males showed increased MLCL levels and increased MLCL-to-cardiolipin (MLCL/CL) ratio compared to WT (Fig. 2E, F), which suggests an impairment in MLCL-AT and cardiolipin remodeling. LCHADD mice of both sexes showed higher oxidized cardiolipin species, including 72:8 (four linleooyl side chains) and oxidized 72:7 (three linoleoyl and one oleoyl side chains) cardiolipin compared to WT mice (Fig. 2G). This could indicate higher oxidative stress in LCHADD mouse hearts. Overall, this data suggests that the hearts of LCHADD mice do have altered cardiolipin content, composition, oxidized ratio, and, at least in male LCHADD mice, there is evidence of impaired cardiolipin remodeling with an increase in MLCL levels.

### Cardiac tissue expression of cardiolipin biosynthesis and remodeling enzymes in LCHADD mice

**Fig. 3:**
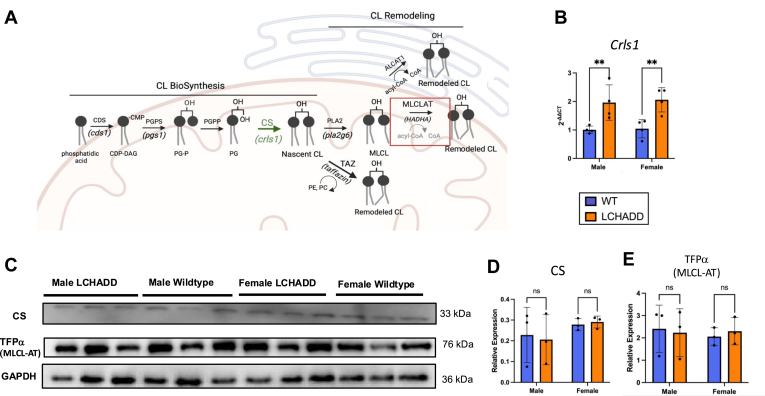
**Differences in cardiolipin synthesis and remodeling pathway in LCHADD mice**. A: Diagram of CL synthesis and remodeling pathways highlighting key enzymes, including MLCL-AT. MLCL-AT is encoded by *Hadha* and may be affected by the c.1528G>C mutation in LCHADD mice. B: mRNA levels of *Crls1* show increased expression in 12-month-old mice of both sexes in LCHADD mice compared to WT (N = 4 per group). C: Western blot of CS and TFPα (MLCL-AT) in individual 11-month-old male and female mice of both genotypes (N = 3 per group). D, E: Densitometric analysis, quantifying CS (D) and TFPα (MLCL-AT) (E) levels relative to GAPDH. Data are presented as mean ± SD with individual data points. Statistics: 2-way ANOVA (genotype & sex) followed by post-hoc Sidak test. ns (not significant), ∗∗ (*P* < 0.01).

To address if other enzymes involved in cardiolipin biosynthesis or remodeling pathways are affected by LCHADD genotype, we examined the levels of mRNA and proteins involved (Fig. 3A). The expression of *Crls1*, which encodes cardiolipin synthase (CS), was elevated in LCHADD mice compared to WT in both sexes (Fig. 3B). However, no difference was found in CS protein levels (Fig. 3C, D and supplemental Fig. S3) (35). Otherwise, we did not see differential expression in other enzymes of cardiolipin synthesis and remodeling pathways (supplemental Fig. S1), including *Hadha* gene expression (Fig. 1A) or MLCL-AT (TFPα) protein expression (Fig. 3C, E and supplemental Fig. S3), which was expected based on previous findings (25). Overall, this data suggests that differences in the cardiolipin profile seen in LCHADD mouse hearts is not due to compensation or changes in protein levels of other enzymes involved in cardiolipin biosynthesis or remodeling.

### Lipid accumulation and composition in LCHADD hearts

**Fig. 4:**
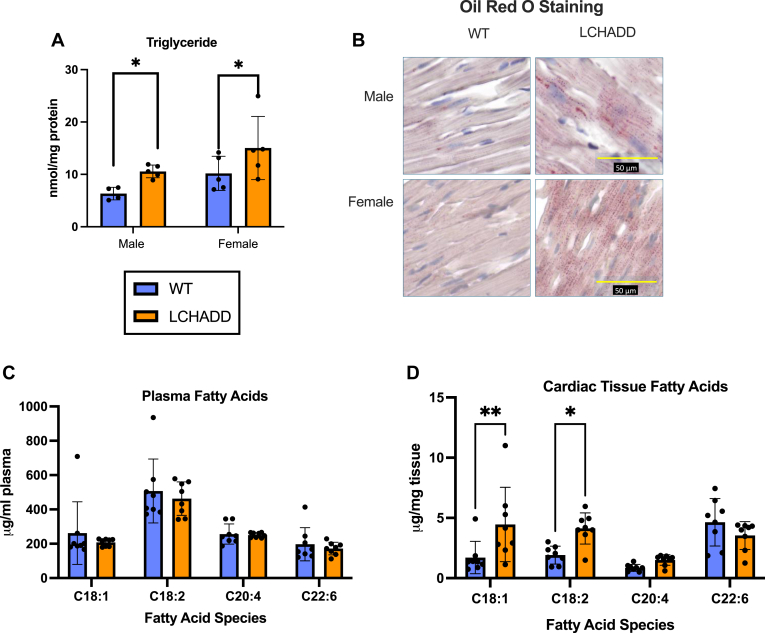
**Concentrations of lipids in serum and cardiac tissue**. A: Heart triglyceride levels were higher in LCHADD compared to WT mice in both males and females (male WT N = 4, all other groups N = 5). B: Representative images of Oil Red O staining of WT and LCHADD heart tissue demonstrate greater lipid droplet accumulation (stained in red) in LCHADD, in both sexes. All images are at equal magnification, yellow bar = 50 μm. C: Serum concentrations of oleic (C18:1), linoleic (C18:2), arachidonic (C20:4), and docosahexaenoic (C22:6) acids were not different between WT and LCHADD mice (N = 8 per group). D: In contrast, cardiac tissue oleic acid and linoleic acid concentration was higher in LCHADD compared to WT mice (N = 8 per group). Data presented as mean ± SD with individual data points. Statistics: 2-way ANOVA with genotype and sex or species as the two factors. Post-hoc Sidak test identified differences between WT and LCHADD are denoted by asterisks: ∗(*P* < 0.05), ∗∗(*P* < 0.01).

Decreased ability to oxidize fatty acids in the mitochondria due to impaired FAO has been shown to cause an accumulation of lipids in tissues of related mouse models (36). To test our LCHADD mice, we measured total tissue triglycerides in 12-month-old LCHADD mouse hearts. Both male and female LCHADD mice had greater triglyceride concentrations than WT counterparts (Fig. 4A). This was supported by Oil Red O staining, which shows that the myocardium of 12-month-old LCHADD mice exhibits more and larger lipid drops compared to WT (Fig. 4B).

Previous research has shown that the tissue availability of fatty acids can influence the cardiolipin composition (37), so we examined the fatty acid profile using GC-MS in both serum and cardiac tissue. Specifically, we measured the essential fatty acids crucial to cardiac cardiolipin's structural integrity: linoleic acid (LA; 18:2), oleic acid (OA; 18:1), arachidonic acid (AA; 20:4), and docosahexaenoic acid (DHA; 22:6). For these species, circulating fatty acids were similar between LCHADD and WT mice regardless of sex (Fig. 4C; Supplemental Fig. 2A). Since plasma fatty acids are primarily a reflection of dietary intake and all mice were fed a similar chow containing both omega-6 and omega-3 fatty acids, it is not surprising that the plasma levels of fatty acids were similar between genotypes.

Interestingly, we observed marked differences in the fatty acid profiles of myocardial tissue. Specifically, LCHADD mice showed a pronounced increase in concentration of OA and LA but no difference in DHA suggesting an abundance of OA and LA in the tissue (Fig. 4D and supplemental Fig. S2B). The myocardial fatty acid profile mirrors the shift in cardiolipin profile, suggesting that the lipid environment in the heart may be significantly influencing the cardiolipin profile and the fatty acid species incorporated into cardiolipin moieties.

### Mitochondrial structure and function

**Fig. 5:**
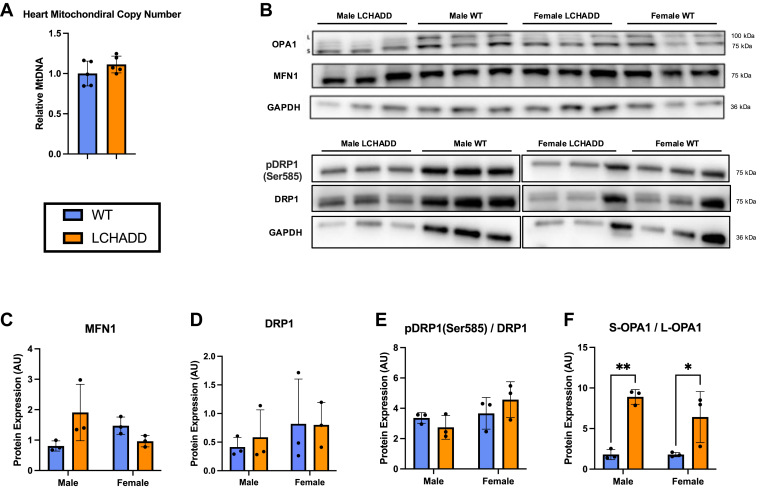
**Mitochondrial copy number and dynamics in LCHADD and WT mice.** A: Relative mitochondrial DNA (mtDNA) copy number in the hearts of 12-month-old LCHADD and WT mice were not different (N = 5 per group). B: Representative Western blots of indicated proteins in 12- to 15-month-old male and female LCHADD and WT mice hearts (N = 3 per group) illustrating alterations in S-OPA1/L-OPA1 ratio. C–F: Densitometric analysis of Western blot showing mitofusin-1 (MFN1), dynamin-related protein-1 (DRP1) and the ratio of phosphorylated (pDRP1) to total DRP1, and optic atrophy-1 (OPA1) as the ratio of short to long isoforms (S-OPA1 and L-OPA1). Relative levels to GAPDH. Data are presented as mean ± SD. Statistical significance indicated by asterisks: ∗ (*P* < 0.05), ∗∗ (*P* < 0.01).

Alterations in cardiolipin composition have been associated with increases in oxidative stress and loss of cristae (38). Anomalies in cardiolipin adversely affect mitophagy and mitochondrial fusion (38, 39), impacting mitochondrial structure and function. Since LCHADD mice have increased oxidized cardiolipin species in the heart, suggesting increased oxidative stress (Fig. 2G), we investigated if observed changes in cardiolipin composition were associated with changes in mitochondrial structure and mitochondrial dynamics. First, mitochondrial copy number was determined to see if LCHADD hearts have higher number of mitochondria, leading to increased total cardiolipin in LCHADD mice shown in Fig. 2D. We examined the ratio of a nuclear gene (*Polb*) to a mitochondrial gene (*mt-Nd6*) and found no difference between genotypes, suggesting similar mitochondrial numbers (Fig. 5A). Next, to explore the possibility of altered fission/fusion machinery, we investigated the protein levels of Mitofusin-1 (MFN1), Optic Atrophy-1 (OPA1), Dynamin-related protein-1 (DRP1), and Ser585-phosphorylated DRP1 (pDRP1; Fig. 5B–F and supplemental Fig. S3). MFN1 and OPA1 are mitochondrial outer- and inner-membrane fusion proteins, respectively, while the ratio of pDRP1 to total DRP is associated with mitochondrial fission (40). There was no significant difference in the protein levels of MFN1 or the pDRP1/total DRP1 ratio (Fig. 5B–E). However, OPA1 showed a significant difference in cleaved forms. The long form, L-OPA1, in LCHADD mice was significantly diminished compared to the short form, S-OPA1 (Fig. 5B, F), indicating higher cleavage of OPA1.

**Fig. 6:**
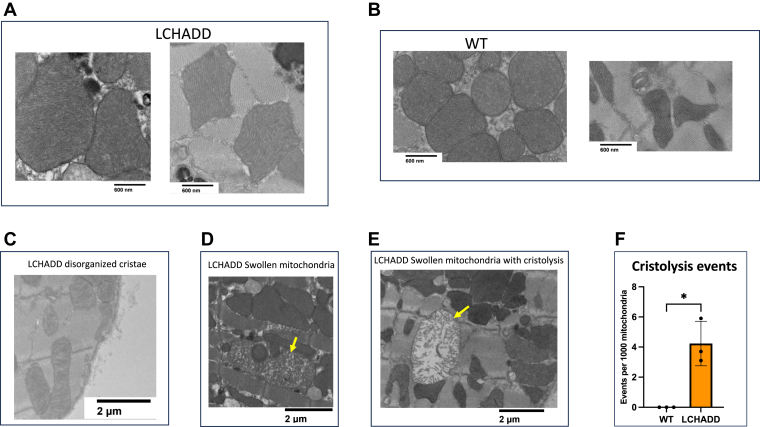
**TEM of LCHADD and WT cells show various states of LCHADD cardiac mitochondrial health**. A, B: LCHADD and WT show healthy mitochondria. C–E: However, LCHADD mitochondria also show mitochondria with disorganized cristae (C), swollen (D), and swollen with cristolysis (E). Swollen mitochondria are marked with arrows. F: Cristolysis is only seen in LCHADD mitochondria.

Higher cleavage of OPA1 is associated with higher fission and altered mitochondrial structure (41). Therefore, we examined cardiac mitochondrial structure with transmission electron microscopy (TEM; Fig. 6). Overall, mitochondrial structure seemed intact. General LCHADD mitochondrial properties, such as mitochondrial density (calculated by mitochondrial number or by mitochondrial area), size, and circularity, were equivalent to WT (supplemental Fig. S4). However, mitochondria from LCHADD tissue showed a wide range of mitochondrial health. Mitochondria with tight, laminar cristae similar to WT could be seen, as well as mitochondria with disorganized cristae. Swollen mitochondria with and without cristolysis were also identified (Fig. 6A–E). Orientation and staining aberrations in many mitochondria prevented the assessment of cristae for both LCHADD and WT. Anecdotally, identified events of disorganized cristae were higher in LCHADD hearts but were a very small percentage, below 0.5%, of total mitochondria (data not shown). However, the presence of swollen mitochondria with cristolysis, which is more apparent, was only seen in LCHADD animals at a rate around 0.4% (Fig. 6F). Altered mitochondrial proteins and structure suggest that further study of mitochondrial dynamics in the LCHADD mouse is warranted.

## Discussion

The development of LCHADD cardiomyopathy has been hypothesized to be related to energy deficiency due to the block in FAO; however, a new non-canonical function of TFPα, the affected protein, has been recently identified. TFPα remodels cardiolipin through MLCL-AT activity which may link LCHADD to cardiolipin metabolism. Previous studies demonstrate that cardiolipin is crucial for maintaining mitochondrial structure and function (42), and alterations in cardiolipin species can lead to mitochondrial dysfunction and cardiac pathologies such as heart failure (38). It is currently not known how the common pathogenic variant in LCHADD, c.1528G>C in *HADHA*, affects the MLCL-AT activity and the cardiolipin profile. In this study, we aimed to further characterize the development of the LCHADD cardiomyopathy and to investigate possible molecular mechanisms involved using a mouse model that is homozygous for c.1528G>C and develops hypertrophic cardiomyopathy similar to cardiomyopathy observed in human patients with LCHADD. Specifically, we addressed how the c.1528G>C pathogenic variant influences the heart cardiolipin profile and mitochondrial function. Our results demonstrate that male and female LCHADD mice have a progressive cardiac phenotype developing eccentric hypertrophic cardiomyopathy over time. These mice also display an altered cardiolipin profile that mirrors the altered fatty acid profile in cardiac tissue, but not in plasma, and male LCHADD mice have an increase in MLCL and the MLCL/CL ratio, which may be the result of a decrease in MLCL-AT activity. LCHADD mice show minor changes in mitochondria indicated by a change in the S-OPA1 and L-OPA1 protein ratio and the presence of some mitochondria with an abnormal structure as shown on TEM images. These findings suggest the possibility that mitochondrial dysfunction due to a change in cardiolipin profile may play a role in the development of LCHADD cardiomyopathy.

LCHADD mice had an increased fraction of tetralinoleoyl cardiolipin and other cardiolipins containing 18-carbon fatty acyl chains, increased cardiolipin species containing AA and other 20-carbon fatty acids, and a decrease in cardiolipin species containing DHA and other 22-carbon fatty acids. We found that LCHADD cardiac tissue displays a fatty acid profile that mirrors the composition of cardiolipin acyl groups. Previous studies have shown that the lipid environment, both in composition and quantity, can significantly influence the cardiolipin profile (37, 43). Mice with lipid accumulation in the liver, also known as hepatic steatosis, had a higher cardiolipin content in the liver (43). This is particularly relevant because lipid accumulation, caused by a loss of FAO, has been commonly seen in the livers and hearts of several different FAO disorder models, including in this study (36, 44, 45, 46, 47, 48). Therefore, the accumulation of specific lipids due to diminished FAO activity may be responsible for the shift in cardiolipin composition in LCHADD hearts.

Although it is speculated, based on the crystal structure, that the LCHADD c.1528G>C variant affects the MLCL-AT activity of TFPα as well as the LCHAD activity, the effect of the c.1528G>C pathogenic variant on cardiolipin remodeling has not been well studied. This is highlighted by the differences in cardiolipin profiles between heterozygous TFPα knockout mice (TFPα^+/−^) and LCHADD in vitro models (24, 49). In this study, we report an increase in the MLCL/CL ratio in 12-month-old LCHADD male mice, which may indicate a destabilization of cardiolipin as it is seen in mouse models of Barth Syndrome; however, LCHADD females do not exhibit the increased ratio. In addition, we observed similar gene expression in the cardiolipin biosynthesis and remodeling pathway enzymes, suggesting no overall change in enzyme expression. We also observed an increase in total cardiac cardiolipin in female LCHADD mice, suggesting increased cardiolipin production. Miklas *et al.* also observed an accumulation of fatty acids in iPSC-derived cardiomyocytes from LCHADD patient fibroblasts, including oleic and linoleic acids, along with altered cardiolipin profiles with decreased C20:2 fatty acids; however, the shift in cardiolipin profiles could be related to the change in fatty acid profiles with the accumulation of media fatty acids (24). In contrast, the TFPα^+/−^ mouse, which has reduced TFPα protein, had a normal plasma fatty acid profile and no shift in cardiolipin species, but there was a decrease in total cardiolipin levels in the heart (49). Our LCHADD mouse model with the c.1528G>C pathogenic variant does not have reduced TFPα protein and we did not observe decreased cardiolipin in the LCHADD mouse like the TFPα^+/−^ mouse. Therefore, we speculate that the c.1528G>C variant may not impair MLCL-AT activity of TFPα protein, but rather it appears the surrounding lipid environment influences the change in the cardiolipin profile; however, a direct measurement of MLCL-AT activity in LCHADD and WT tissues is needed to confirm this.

Cardiolipin has been shown to play an important role in the formation of the cristae and, subsequently, the organization of the electron transport chain supercomplexes (42). Disruption of the supercomplex formation can enhance reactive oxygen species (ROS) production, causing oxidative damage to the cells and increase turnover of cardiolipin as indicated by increased levels of MLCL (50). In this study, LCHADD mice had increased oxidized cardiolipin species, suggesting increased oxidative stress. Altered OPA1 isoform ratios and the presence of a few swollen mitochondria with abnormal cristae exclusively in LCHADD hearts suggest some dysfunctional mitochondria. While S-OPA1 and L-OPA1 are thought to work in concert to maintain cristae structure, S-OPA1 by itself can maintain cristae structure and mitochondrial energetics during oxidative stress (51). It is unknown if the altered cardiolipin in LCHADD hearts is related to the increase of S-OPA1 levels or if either is involved in mitochondrial fragmentation and dysfunction with increased oxidative stress. How increased oxidative stress and loss of cristae ultimately contribute to cardiomyopathy will need to be studied further.

Surprisingly, we observed some sex-specific differences among LCHADD mice over time. For example, LCHADD male mice appeared to develop some cardiac dysfunction as early as 3-months of age, whereas female cardiac function at the same age was similar to WT. However, both male and female mice exhibited cardiac dysfunction at 12 months of age compared to WT. The cardiolipin profile also differed between 12-month-old male and female mice in that male LCHADD mice displayed an accumulation of MLCL whereas female LCHADD mice showed an increase in total cardiolipin levels. The sex-specific differences in the cardiolipin profile may be contributing to the different timing of cardiac dysfunction, but how they are related is not currently known. It is important to note that sex-specific differences have not been previously reported among LCHADD patients. However, we recently reported that male patients with LCHADD deficiency have an increased risk of a sudden cardiac death or arrest compared to female patients (9). Determining if these sex differences are species-specific will be important in determining the applicability of these findings to patients.

One limitation of this study was that we did not measure the cardiolipin profile and mitochondrial structures at multiple time points; therefore, we cannot definitively say that changes in cardiolipin profiles precede or are responsible for altering the mitochondrial structure. Nor can we connect mitochondrial dysfunction to the development of cardiomyopathy. Evaluating the cardiolipin profile and mitochondrial structure in mice prior to the development of cardiac dysfunction will be crucial in providing evidence for causality versus correlation. Another potential limitation is using sums of cardiolipin species to compare groups as there is some inherent inaccuracy in the quantification between various species when using one internal standard in the assay for quantification.

In conclusion, cardiolipin profiles are altered in the cardiac tissue of LCHADD mice, and the alterations in acyl composition is similar to the alterations in the composition of accumulated fatty acids in the heart. LCHADD mice did not show a reduction in cardiolipin. Collectively, we suggest that these differences in cardiolipin species are related to the accumulation of lipids in the heart tissue due to impaired FAO and not specific to the MLCL-AT activity of TFPα. The cardiac tissue has a similar mitochondrial number but an altered S-OPA1/L-OPA1 ratio and the presence of swollen mitochondria with disorganized cristae on TEM, which was absent in WT mice. The causal relationship between altered cardiolipin profiles, changes in mitochondrial structure, and the decreased cardiac function warrants further investigation.

## Data availability

All data is contained within the manuscript.

## Supplemental data

This article contains supplemental data (28, 52).

## Conflicts of interests

The authors declare the following financial interests/personal relationships which may be considered as potential competing interests: Melanie B. Gillingham has received speaker honorarium from Ultragenyx Pharmaceutical Inc., Vitaflow, and Nutricia, and received research grant funds from Nestle Health Science and Reneo Pharmaceutical.

## Supplementary data

Supplemental Tables and Figures

Data transparency
