# Supplementary material for: Cardiomyopathy in a c.1528G>C Hadha mouse is associated with cardiac tissue lipotoxicity and altered cardiolipin species
Source: J Lipid Res. 2025 Mar 29;66(5):100792. doi: 10.1016/j.jlr.2025.100792 (PMC12084506; doi:10.1016/j.jlr.2025.100792)
Supplement: Supplemental Tables and Figures [file mmc1.pdf]

Supplemental Tables and Figures for Cardiomyopathy in a c.1528G>C *Hadha* Mouse is associated with Cardiac Tissue Lipotoxicity and Altered Cardiolipin Species

Supplemental Table 1: qPCR primers for cardiolipin synthesis and remodeling enzymes.

Gene	Forward primer	Reverse primer
Cds1[a]	5'-CGCACGTCACTTTACTGATA-3'	3'-GAATCCTTCCCAAGTCTTCT
Pgs1	5'-TTATTGGCTCCCTTGCTGTC-3'	5'-TGTCCCCAAGTAGAGGGATG-3'
Crls1[b]	5'-GGGCTACCTGATTCTTGAAGA -3'	5'-GGCCCAGTTTCGAGCAATAA-3'
Pla2g6	5'-GCCAACAGCACAGAGAATGA-3'	5'-TCAGCCCTTGGTTGTTTACC-3'
Alcat1	5'-CATGCAAGTTGCGGCCTTTA-3'	5'-CTGAAGTCCGTTCTTCTCAG-3'
Hadha	5'-ATGGCGTCAAAGGGGATGTGGC-3'	5'-TGGTCGTTGGCCCAGATTTCGT-3'
Hadhb	5'-CTCAACACCACCAGCCACGACG-3'	5'-CCCTGGGAGCTGGCTTCTCTGA-3'
Acadvl	5'-GCGGACGGCGCCATTGACCTCTATG-3'	5'-ATCCGAGTTGCAGCCTCAATGCA-3'
Taffazin	5'-AACTCCGCCACATCTGGAAC-3'	5'-GCGCAGGAACTCAGAACTC-3'
B-Actin	5'-CCCGGGCTGTATTCCCCTCCAT-3'	5'-TGGGCCTCGTCACCCACATAGG-3'

[a]Previously published in: Qi Y, et al. CDP-diacylglycerol synthases regulate the growth of lipid droplets and adipocyte development. J Lipid Res. 2016 May;57(5):767-80.
[b]Previously published in: Sustarsic EG, et al. Cardiolipin Synthesis in Brown and Beige Fat Mitochondria Is Essential for Systemic Energy Homeostasis. Cell Metab. 2018 Jul 3;28(1):159-174.e11.

Supplemental Table 2: Antibody concentrations

**Protein**	**Primary Source**	**Primary Antibody Concentration**	**Secondary Antibody Concentration**
TFPα	Invitrogen PA527348	1:1000	1:10000
TFPβ	Invitrogen PA5-117024	1:1000	1:10000
VLCAD	Invitrogen PA5-29959	1:1500	1:10000
CRLS	Invitrogen PA5-25338	1:500	1:2000
MFN1	Proteintech 13798-1-AP	1:1000	1:5000
DRP1	Cell Signaling Technology 14647T	1:1000	1:5000
pDRP1	Invitrogen PA5-64821	1:1000	1:10000
OPA1	Proteintech 27733-1-AP	1:1000	1:5000
GAPDH	Proteintech HRP-60004 or 60004-1-IG	1:2000	1:5000

Supplemental Table 3: Primers used to calculate mitochondrial copy number

	Forward primer	Reverse Primer
Mt-Nd6	CACCCAGCTACTACCATCATTC	GTTTGGGAGATTGGTTGATGTATG
Polb	GGCGGATGGTGTACTCATT	ACTGTGGTGTTCTCTACTTCAC

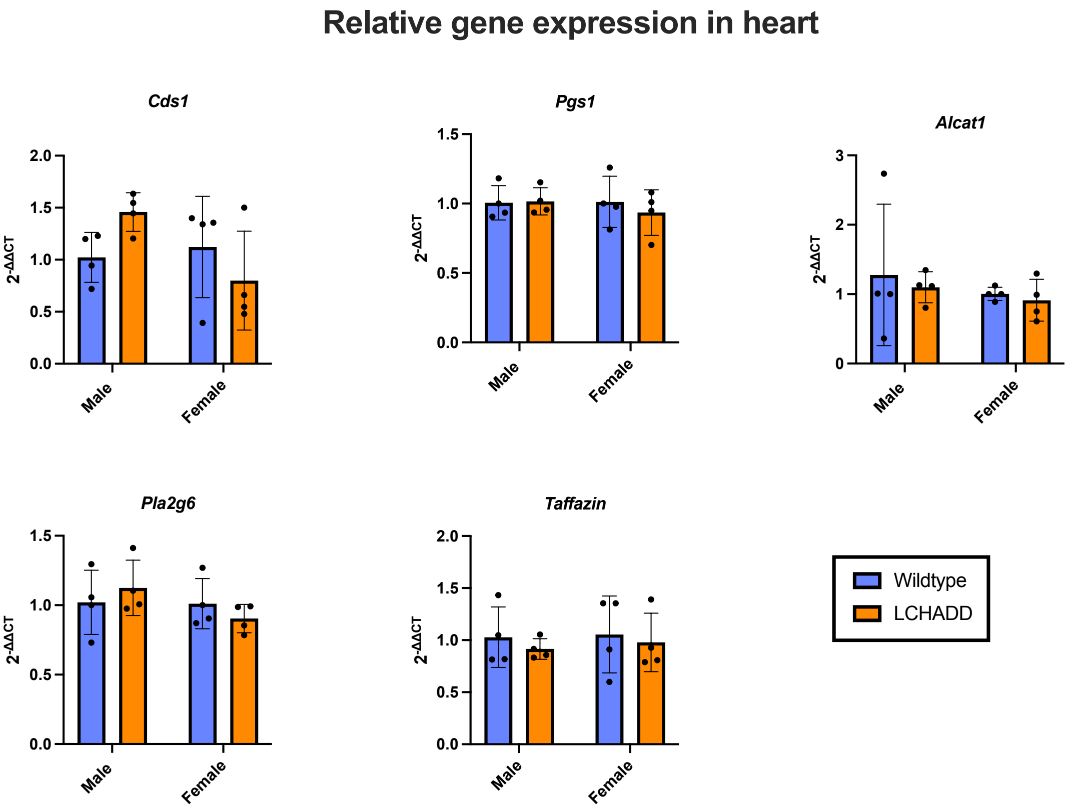

Supplemental Figure 1: Cardiolipin Gene Expression. The relative gene expression levels of cardiac-related genes (*Cds1, Pgs1, Alcat1, Pla2g6, Taffazin*) across male and female mice. Expression levels are normalized to beta actin and quantified using the ΔΔCT method. Data presented as mean ± SD with individual data points. Statistical significance was assessed by two-way ANOVA; no differences identified at $p<0.05$.

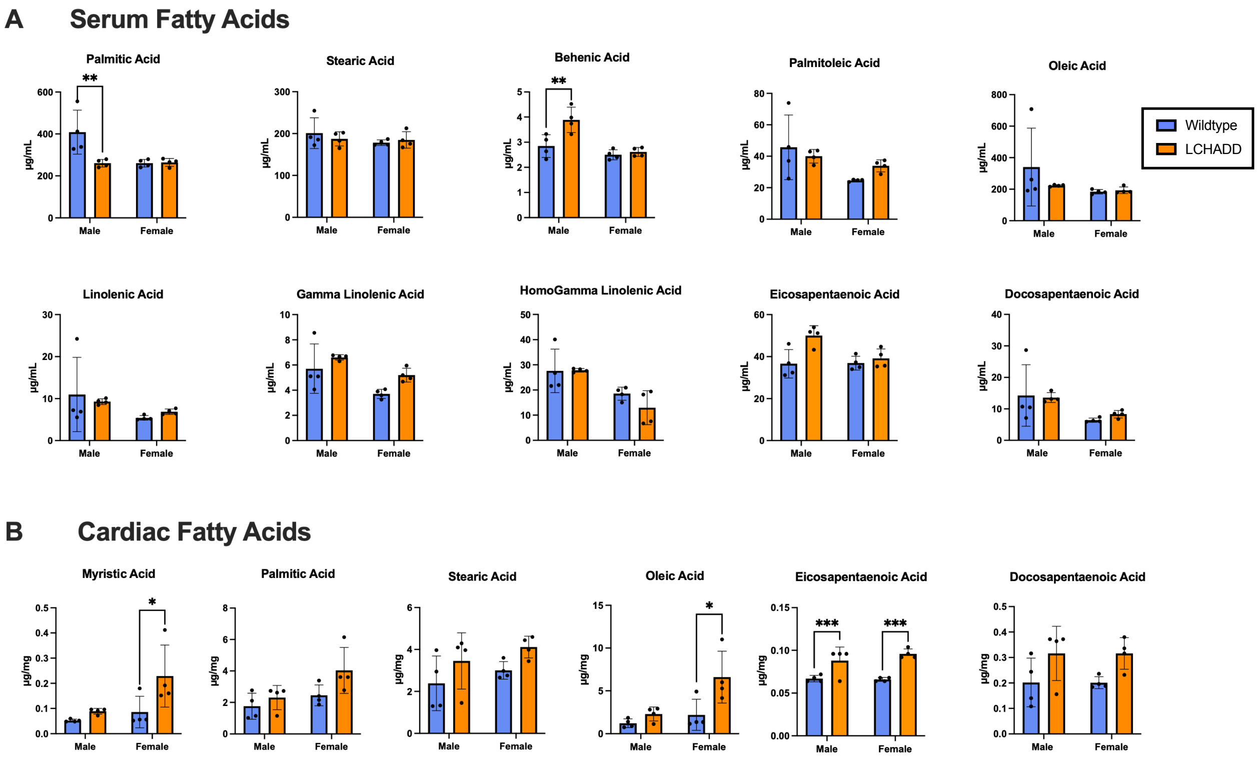

Supplemental Figure 2: Serum and Cardiac Fatty Acid Profiles in WT and LCHADD Mice by Sex. (A) Mean concentrations (µg/mL) of palmitic, stearic, behenic, palmitoleic, oleic, linolenic, gamma-linolenic, homogamma linolenic, eicosapentaenoic, and docosapentaenoic acids in serum of male and female mice, across genotypes: LCHADD and WT. (B) Cardiac tissue fatty acid concentrations (µg/mg) for myristic, palmitic, stearic, oleic, eicosapentaenoic, and docosapentaenoic acids. Data expressed as mean ± SD with individual data points. Statistical analysis was assessed by two way-ANOVA with Sidak's multiple comparison post-hoc test. Significant differences denoted by asterisks: * ($P<0.05$), ** ($P<0.01$), *** ($P<0.001$), ($N=4$).

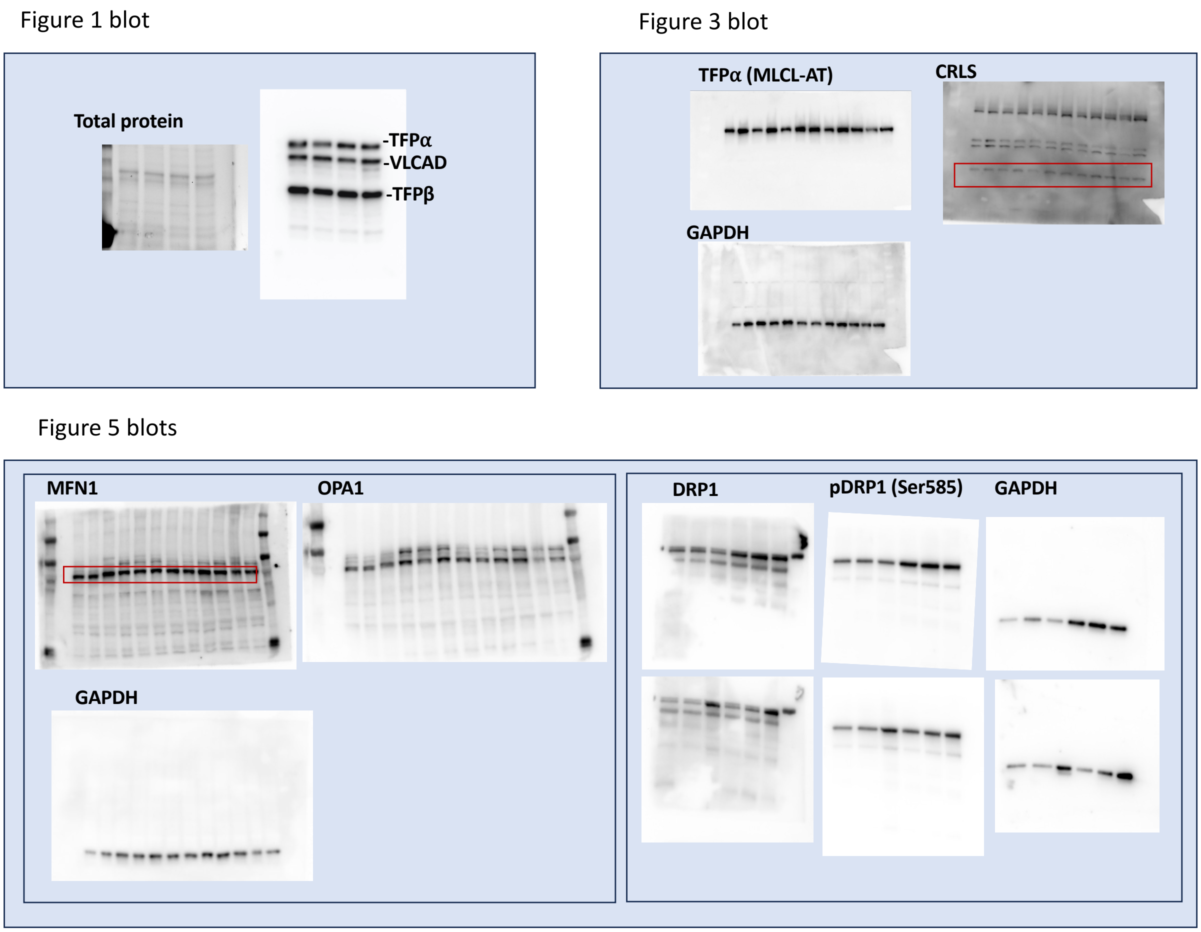

Supplemental Figure 3: Western blots. The full western blots illustrated in Figures 1, 3, and 5 are provided.

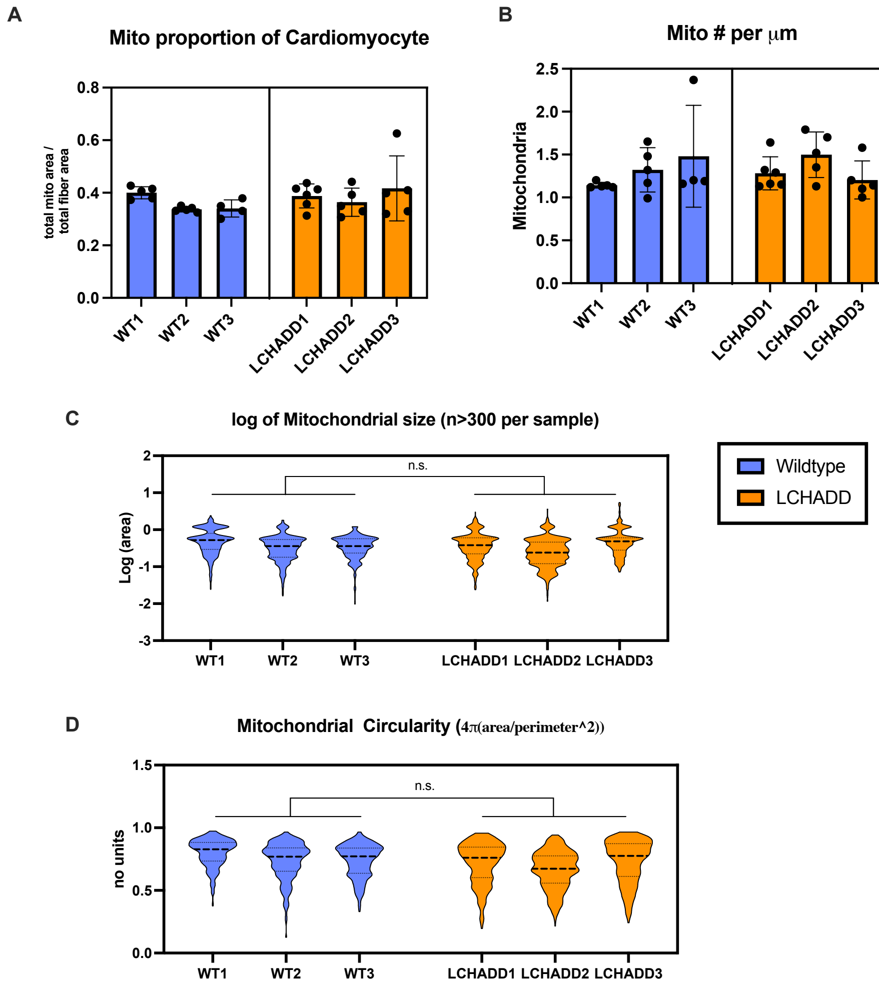

Supplemental Figure 4: Quantification of cardiac mitochondrial properties. Mitochondria from multiple TEM images of 12-month-old WT and LCHADD males (n=3 mice per genotype; 4-6 images per mouse) were analyzed. Density of the mitochondria was assessed as a proportion of area covered (A) and number of mitochondria per square µm (B). Mitochondria size (C) and circularity (D) is also reported. Mitochondrial size was transformed for analysis using a log function, graphed here. Data with error bars is expressed as mean ± SD. For violin pot data, bold and lighter dotted lines represent medians and quartiles, respectively. All data was analyzed by nested T-tests. No significant differences noted.
